# Supplementary material for: Cis‐nonProline peptides: Genuine occurrences and their functional roles
Source: Protein Sci. 2025 May 24;34(6):e70157. doi: 10.1002/pro.70157 (PMC12102755; doi:10.1002/pro.70157)
Supplement: Supplementary file 1 — Table S1: Is a pdf for human readability, including all genuine cis‐nonPro cases used here, with PDB code, residue numbers and amino acids, resolution, number of cis‐nonPro, abbreviated code, whether in CAZy, tertiary structure, molecule name, notes, CAZy family, and omega. [file PRO-34-e70157-s003.pdf]

residue 1	residue 2	reso-lution	# cnP	GG, Wx, ..	CAZy	tertiary struct	molecule	notes	CAZy family	omega
CAZy										
1czf A 228 GLY	1czf A 229 SER	1.68			y	b-helix	endopolygalacturonase-2		GH28	0.08
1e0w A 81 HIS	1e0w A 82 THR	1.2			y	TIM	xylanase A	str 3	GH10	4.39
1e4m M 457 TRP	1e4m M 458 ALA	1.2		Wx	y	TIM	myrosinase S-glycosidase	str 8, long lp	GH1	-5.75
1edq A 190 GLY	1edq A 191 PHE	1.55	3		y	TIM	chitinase A, Serratia marcescens	str 2	GH18	0.26
1edq A 315 GLU	1edq A 316 PHE	1.55	3		y		"	str 4	"	-3.69
1edq A 539 TRP	1edq A 540 GLU	1.55	3		y		"	str 8	"	-6.7
1gai A 23 GLY	1gai A 24 ALA	1.7			y	1.50.10.10	glucoamylase		GH15	0.9
1goi B 144 GLU	1goi B 145 TYR	1.45	3		y	TIM	chitinase B, Serratia marcescens	str 4, 4th cnP not good	GH18	8.08
1goi B 403 TRP	1goi B 404 HIS	1.45	3	Wx	y		"	str 8	"	-10.89
1goi B 50 SER	1goi B 51 PHE	1.45	3		y		"	str2	"	2.15
1gqL B 525 GLN	1gql B 526 GLY	1.67			y	TIM	alpha-glucuronidase		GH67	0.79
1h1n A 273 TRP	1h1n A 274 ALA	1.12	2	Wx	y	TIM	endogluconase T aurant		GH5	5.15
1h1n A 288 PRO	1h1n A 289 ASP	1.12	2		y		"		"	-4.38
1h5v A 262 TRP	1h5v A 263 SER	1.1		Wx	y	TIM	endoglucanase Cel5A	end str 8, long lp	GH5	1.53
1hjs B 147 THR	1hjs B 148 GLU	1.87			y	TIM	beta-1,4-galactanase		GH53	2.33
1itx A 204 GLU	1itx A 205 TYR	1.1	3		y	TIM	chitinase A1, bacterial	str 4	GH18	6.15
1itx A 433 TRP	1itx A 434 GLU	1.1	3	Wx	y		"	str 8, act site	"	-2.14
1itx A 76 ALA	1itx A 77 PHE	1.1	3		y		"	str 2	"	4.29
1jnd A 295 PRO	1jnd A 296 VAL	1.3	3		y	TIM	imaginal-disk Growth F	str 4, chitinase-like	GH18	0.76
1jnd A 39 GLY	1jnd A 40 TYR	1.3	3		y		"	str 2	"	-0.84
1jnd A 394 PHE	1jnd A 395 ASP	1.3	3		y		"	str 8	"	-11.8
1k5c A 200 GLY	1k5c A 201 SER	0.96	2		y	b-helix	polygalacturonidase	binds substrate	GH28	13.63
1k5c A A 331 GLY	1k5c A A 332 GLY	0.96	2	GG	y		"	alt b is the cis, gd ed	"	-0.85
1k6a A 83 HIS	1k6a A 84 THR	1.14			y	TIM	xylanase		GH10	-2.79
1kfw A 192 GLU	1kfw A 193 TRP	1.74	3		y	TIM	chitinase B	str 4	GH18	-0.13
1kfw A 412 TRP	1kfw A 413 GLU	1.74	3	Wx	y		"	str 8	"	-1.05
1kfw A 54 SER	1kfw A 55 PHE	1.74	3		y		"	str 2	"	0.13
1kwg A 350 PHE	1kwg A 351 ARG	1.6			y	TIM	beta-galactosidase		GH42	1.17
1nof A 200 VAL	1nof A 201 ALA	1.42			y	TIM	beta-1,4-xylanase	str 5	GH30	-8.72
1ofL A 293 GLY	1ofl A 294 GLY	1.7		GG	y	b-helix	chondroitin B lyase		PL6	6.72
1om0 A 256 TRP	1om0 A 257 ASP	1.8	2	Wx	y	TIM	xylanase inhibitor	classic, but inactive	GH18	-0.71
1om0 A 36 SER	1om0 A 37 PHE	1.8	2		y		"		"	0.34
1qw9 B 350 ALA	1qw9 B 351 GLN	1.2	2		y	TIM	arabinofuranosidase	cnPs great in ch B	GH51	1.25
1qw9 B 72 GLY	1qw9 B 73 GLY	1.2	2	GG	y		"	THE dire Trp dire Lys,Met,Ile	"	7.18
1rh9 A 360 TRP	1rh9 A 361 GLN	1.5		Wx	y	TIM	tomato b-mannanase		GH5	-0.4
1tvn B 256 TRP	1tvn B 257 ALA	1.41		Wx	y	TIM	cellulase Cel5G		GH5	0.34
1ug6 A 385 TRP	1ug6 A 386 SER	0.99		Wx	y	TIM	T therm b-glucosidase	end str 8, act	GH1	12.72
1uqz A 104 HIS	1uqz A 105 THR	1.55			y	TIM	xylanase C	str 3	GH10	-2.72
1uuq A 376 TRP	1uuq A 377 ALA	1.5		Wx	y	TIM	exo-mannosidase		GH5	3.43
1uws B 425 TRP	1uws B 426 SER	1.95		Wx	y	TIM	GH1 b-glycosidase		GH1	1.62
1v0z B 332 THR	1v0z B 333 SER	1.84			y	b-propeller 6	duck-virus neuraminidase		GH34	4.5
1vbu A 598 HIS	1vbu A 599 THR	1.8			y	TIM	flu virus neuraminidase	xylanase B ? Str 3	GH10	-1.72
1w3h A 79 HIS	1w3h A 80 THR	1.5			y	TIM	xylanase A		GH10	15.37
1w9p A 177 GLU	1w9p A 178 TYR	1.7	3		y	TIM	chitinase, Aspergillus fumigatus	str 4	GH18	0.41
1w9p A 384 TRP	1w9p A 385 ASP	1.7	3	Wx	y		"	str 8	"	-5.12
1w9p A 75 ALA	1w9p A 76 PHE	1.7	3		y		"	str 2	"	-3.63
1wcg B 416 TRP	1wcg B 417 SER	1.1		Wx	y	TIM	aphid myrosinase		GH1	6.13
1wd3 A 176 CYS	1wd3 A 177 CYS	1.75		vicSS	y	greek key	arabinofuranosidase	& CBM42	GH54	-1.02
1wdr A 419 LEU	1wdr A 420 ARG	1.35			y	TIM	b-amylase	str 8, Arg act site	GH14	0.23
1x1i A 145 TYR	1x1i A 146 ASN	1.8			y	1.50.10.10	xanthan lyase		PL8	-0.32
1x38 A 206 LYS	1x38 A 207 HIS	1.7	2		y	TIM	b-D-glucan exohydrolase	K, H at act site	GH3	-5.76
1x38 A 208 PHE	1x38 A 209 VAL	1.7	2		y		"	NB: 2 cnP 2 apart	"	-6.15
1yq2 C 147 GLU	1yq2 C A 148 SER	1.9	altA		y	TIM	cold-active b-galactosidase	str 2	GH2	-2.41
1yq2 C 368 SER	1yq2 C 369 HIS	1.9	3		y		"	str 4	"	-1.69
1yq2 C 552 TRP	1yq2 C 553 GLU	1.9	3	Wx	y		"	str 8, Trp id'ed as act site	"	-10.09
2c0h A 337 TRP	2c0h A 338 SER	1.6	2		y	TIM	mussel b-mannanase	str 8	GH5	-15.2
2c0h A 40 GLN	2c0h A 41 ALA	1.6	2		y		"	str 2	"	-18.46
2cks A 389 TRP	2cks A 390 ASN	1.6		Wx	y	TIM	T fusca endoglucanase Cel5A		GH5	-0.62
2cn3 B 161 GLY	2cn3 B 162 GLU	1.95	2		y	b-propeller 7	xyloglucanase		GH74	-1.1
2cn3 B 348 MET	2cn3 B 349 ASN	1.95	2		y		"		"	-5.01
2cyg A 309 PHE	2cyg A 310 GLU	1.45			y	TIM	beta-1,3-glucanase		GH17	5.12
2d1z A 81 HIS	2d1z A 82 THR	1.6			y	TIM	beta-1,4 xylanase	& CBM13	GH10	-0.28
2d73 A 229 THR	2d73 A 230 ALA	1.6			y	2x 8-strand	glucosidase/glucoamylase		GH97	-10.65
2ddx A 16 GLY	2ddx A 17 GLN	0.86			y	TIM	xylanase	end str 1 in TIM barrel	GH26	4.44
2dep A 121 HIS	2dep A 122 THR	1.8			y	TIM	xylanase B	str 3	GH10	-0.52
2e3z B 415 TRP	2e3z B 416 SER	1.5		Wx	y	TIM	fungal b-glucosidase		GH1	1
2e9m A 417 TRP	2e9m A 418 SER	1.8		Wx	y	TIM	b-glycosylceramidase		GH1	10.71
2eab A 565 PRO	2eab A 566 GLU	1.12	2		y	hlx barrel	fucosidase	E566 catalytic general acid	GH95	-7.01

2eab A 760 HIS	2eab A 761 ALA	1.12	2		y		"	both cnPs are in turns	"	-10
2hvm A 255 TRP	2hvm A 256 SER	1.8	2	Wx	y	TIM	chitinase/lysozyme, plant	str 8	GH18	-0.05
2hvm A 31 ALA	2hvm A 32 PHE	1.8	2		y		"	str 2	"	12.4
2je8 B 115 ASP	2je8 B 116 THR	1.7	2		y	TIM	B thetaio b-mannanase		GH2	-1.75
2je8 B 645 TRP	2je8 B 646 GLN	1.7	2		y		"		"	3.55
2jjb A 312 ASP	2jjb A 313 PHE	1.9	2		y	1.50.10.10	a-a-trehalase		GH37	-2.63
2jjb A 517 GLY	2jjb A 518 PHE	1.9	2		y		"		"	-7.89
2jkb A 271 SER	2jkb A 272 LYS	1.54			y	b-propeller 6	trans-sialidase B	& CBM40	GH33	2.64
2osx A 382 TRP	2osx A 383 SER	1.1	2	Wx	y	TIM	endo-glycoceramidase		GH5	4.01
2osx A 61 THR	2osx A 62 ALA	1.1	2		y		"		"	-8.14
2oxn A 160 LYS	2oxn A 161 HIS	1.7			y	TIM	N-acetylhexosaminidase		GH3	-0.73
2pc8 A 363 TRP	2pc8 A 364 SER	1.8		Wx	y	TIM	Candida exo-b-glucanase		GH5	8.52
2q8x B 81 HIS	2q8x B 82 THR	1.45			y	TIM	xylanase (intrcellular)		GH10	1.39
2qmj A 181 GLY	2qmj A 182 GLU	1.9	2		y	TIM	maltase/glucoamylase		GH31	-3.15
2qmj A 446 GLU	2qmj A 447 VAL	1.9	2		y		"	~str 4, indirect to act site	"	4.87
2uy2 A 285 TRP	2uy2 A 286 ASP	1.6	2	Wx	y	TIM	endochitinase Sacchar cerevisiae	str 8	GH18	-5.66
2uy2 A 59 SER	2uy2 A 60 PHE	1.6	2		y		"	str 2	"	8.25
2v8i A 246 GLY	2v8i A 247 VAL	1.5			y	b-helix	pectate lyase		PL2	-6.57
2vmi A 956 ALA	2vmi A 957 HIS	1.7			y	jellyroll	alpha-1,2-L-fucosidase	& CBM51	GH95	-12.52
2vtf B 393 ARG	2vtf B 394 TRP	1.79			y	TIM	N-acetylhexosaminidase		GH85	1.7
2w20 A 776 ALA	2w20 A 777 TYR	1.49			y	b-propeller 6	sialidase A		GH33	-12.76
2w3z A 116 GLY	2w3z A 117 VAL	1.45			y	7strand barrel	polysaccharide deacetylase	CE4	GH4	-3.6
2w61 A 307 TYR	2w61 A 308 MET	1.62			y	TIM	glucanosyltrans'ase &CBM	Y at act site str 8 (+2 wrong)	GH72	0.92
2w91 A 566 ARG	2w91 A 567 TRP	1.4			y	TIM	N-acetylglucosaminidase D		GH85	12.63
2whL A 251 TRP	2whl A 252 SER	1.4		Wx	y	TIM	mannanase	str 8, act site	GH5	-11.95
2wmf A 301 GLU	2wmf A 302 CYS	1.5			y	TIM	beta-galactosidase	LewisY-specific	GH98	-13.63
2wmi B 710 GLU	2wmi B 711 HIS	1.9			y	TIM	fucolectin-rel beta-gal	str 8, gp A/B-specific	GH98	-7.68
2x3h C 153 ASN	2x3h C 154 ASP	1.6			y	b-helix	K5 polysaccharide lyase		PL0	8.24
2y8k A 174 ALA	2y8k A 175 TRP	1.47			y	TIM	xylanase (arabinoxylan)	out in loop str 4	GH5	10.99
2zut C 212 THR	2zut C 213 THR	1.9			y	TIM	lacto-N-biose phosphorylase		GH112	-0.91
3a4w B 447 ALA	3a4w B 448 PHE	1.8	2		y	TIM	chitinase, archaeal	str 2	GH18	-12.41
3a4w B 664 TRP	3a4w B 665 SER	1.8	2	Wx	y		"	str 8	"	0.59
3ahx D 399 TRP	3ahx D 400 SER	1.9		Wx	y	TIM	Clostridium b-glucosidase		GH1	0.83
3ahz A 444 TRP	3ahz A 445 SER	1.34		Wx	y	TIM	termite b-glucosidase		GH1	3.26
3aLf A 115 GLU	3alf A 116 TYR	1.2	3		y	TIM	chitinase V, Nicotiana tobaccum	str 4	GH18	3.5
3aLf A 30 ALA	3alf A 31 PHE	1.2	3		y		"	str 2	"	-4.51
3aLf A 326 TRP	3alf A 327 HIS	1.2	3	Wx	y		"	str 8, act site	"	-6.18
3b9d A 191 GLY	3b9d A 192 PHE	1.72	3		y	TIM	chitinase A, Vibrio harveyi	str 2	GH18	-6.2
3b9d A 315 MET	3b9d A 316 PHE	1.72	3		y		"	str4	"	-4.89
3b9d A 570 TRP	3b9d A 571 GLU	1.72	3	Wx	y		"	str 8	"	-8.48
3bmx B 221 LYS	3bmx B 222 HIS	1.4			y	TIM	N-acetylglucosaminidase		GH3	-4.79
3c7f A 367 GLY	3c7f A 368 ARG	1.55			y	greek key	endo-1,4-b-xylanase, arabinofuranohydrolase ? CBM6		GH43	-19.15
3c7t A 303 GLY	3c7t A 304 LYS	1.76			y	parallel b	ecdysone phosphate phosphatase		"	-4.81
3civ A 20 PHE	3civ A 21 VAL	1.9	2		y	TIM	endo-b-1,4-mannanase		GH113	8.91
3civ A 281 TRP	3civ A 282 GLU	1.9	2	Wx	y		"		"	-0.63
3cmg A 110 ASN	3cmg A 111 SER	1.9	3		y	TIM	b-galactosidase	beta domain	GH2	-3.2
3cmg A 358 ALA	3cmg A 359 HIS	1.9	3		y		"	str 2	"	-6.64
3cmg A 562 TRP	3cmg A 563 ASN	1.9	3	Wx	y		"	str 8	"	5.68
3cmj A 434 TRP	3cmj A 435 SER	1.6		Wx	y	TIM	soil b-glucosidase		GH1	2.25
3dgt A 171 GLU	3dgt A 172 THR	1.5			y	greek key	beta-1,3-glucanase		GH16	11.46
3ebv A 271 TRP	3ebv A 272 SER	1.5	2	Wx	y	TIM	put chitinase A strep	str 8	GH18	-0.43
3ebv A 36 ALA	3ebv A 37 PHE	1.5	2		y		"	str 2	"	4.84
3eju A 405 PHE	3eju A 406 THR	1.32			y	1.20.1270.50	alpha-mannosidase II		GH38	-4.24
3eqn B 205 GLY	3eqn B 206 GLY	1.7	2	GG	y	b-helix	Ph chrys b-glucanase	btw b-hlx strands	GH55	0.99
3eqn B 628 GLY	3eqn B 629 LEU	1.7	2		y		"	Glu 633 act site	"	-19.78
3f5L B 433 TRP	3f5l B 434 SER	1.37		Wx	y	TIM	plant b-glucosidase		GH1	4.01
3fy1 B 140 GLU	3fy1 B 141 TYR	1.7	3		y	TIM	chitinase, human	str 4	GH18	-5.26
3fy1 B 360 TRP	3fy1 B 361 ALA	1.7	3	Wx	y		"	str 8	"	-3.22
3fy1 B 57 ALA	3fy1 B 58 PHE	1.7	3		y		"	str 2	"	-1.16
3gne A 222 GLY	3gne A 223 GLY	1.2		GG	y	greek key	alginate lyase		PL14	-6.36
3gnr A 444 TRP	3gnr A 445 SER	1.81		Wx	y	TIM	rice b-glucosidase		GH1	3.32
3gvL A 330 GLN	3gvl A 331 ASP	1.41			y	b-propeller 6	phage K1Fendosialidase		GH58	-25.01
3gza A 226 TYR	3gza A 227 HIS	1.6			y	TIM	put. Fucosidase		GH29	5.84
3gzk A 342 GLY	3gzk A 343 TRP	1.8			y	1.50.10.10	cellulase		GH9	2.03
3hn3 B 384 SER	3hn3 B 385 HIS	1.7	2		y	TIM	b-glucuronidase	str 2	GH2	-12.75
3hn3 B 587 TRP	3hn3 B 588 ASN	1.7	2	Wx	y		"	str 8	"	5.29
3ian A 321 TRP	3ian A 322 SER	1.75	2	Wx	y	TIM	chitinase A	str 8	GH18	8.39
3ian A 83 SER	3ian A 84 PHE	1.75	2		y		"	str 2	"	6.86
3man A 254 TRP	3man A 255 SER	1.6		Wx	y	TIM	T fusca mannanase		GH5	-7.54
3mmw D 286 TRP	3mmw D 287 GLU	1.85		Wx	y	TIM	T maritima endoglucanase Cel5A		GH5	1.25
3mu7 A 254 TRP	3mu7 A 255 ASP	1.29	2	Wx	y		xylanase/amylase inhibitor	str 8	GH18	7.79

3mu7 A 33 GLY	3mu7 A 34 PHE	1.29	2		y		"	str 2	"	5.45
3n11 A 333 TRP	3n11 A 334 SER	1.35	2	Wx	y	TIM	chitinase A	str 8	GH18	2.96
3n11 A 65 SER	3n11 A 66 PHE	1.35	2		y		"	str 2	"	2.13
3ogr A 342 TYR	3ogr A 343 MET	1.5			y	TIM	beta-galactosidase		GH35	-13.33
3q4s A 119 GLU	3q4s A 120 LEU	1.98			y	parallel b	glycogenin		GT8	-7.77
non-CAZy										
1avb A 82 ALA	1avb A 83 TYR	1.9				lectin	arcelin-1, Phaseolus vulgaris			-0.19
1ax0 A 88 ALA	1ax0 A 89 ASP	1.9				lectin	legume lectin			-0.15
1beh A 82 ARG	1beh A 83 GLU	1.75					P-ethanolamine-bdg prot	lipid-bdg		-7.57
1cru B 324 LEU	1cru B 325 TYR	1.5			?	b-prop	quinoprot glucose DH			6.49
1d3g A 119 GLY	1d3g A 120 SER	1.6	2				dihydroorotate DH	both needed to bind FMN		5.65
1d3g A 282 VAL	1d3g A 283 THR	1.6	2				"			13.39
1es5 A 211 GLY	1es5 A 212 VAL	1.4					D-D peptidase, b-lactamase			4.32
1fny A 86 ALA	1fny A 87 ASP	1.81				lectin	Robinia bark aggluinin	jellyroll		-0.34
1fsg A 78 LEU	1fsg A 79 LYS	1.05				dbl-wd	hX-Gua P-ribosyltransferase	act site, Mg		-3.27
1gnt A 311 ASN	1gnt A 312 CYS	1.25				2 dbl-wd	Fe-S-O cluster protein	Cys bnds cluster		3.36
1gq6 C 118 GLY	1gq6 C 119 GLY	1.75		GG			related to arginase	in clav acid synthesis		0.58
1gte D 734 ALA	1gte D 735 THR	1.65					dihydropyrimidine DH			-0.74
1gwi B 293 LEU	1gwi B 294 ILE	1.92					mono-oxygenase			0
1hgx B 46 LEU	1hgx B 47 THR	1.9					HGX P-ribosyltransferase			0.51
1i0L A 51 LEU	1i0L A 52 LYS	1.72					HGX P-ribosyltransferase			-1.44
1j2r A 140 ILE	1j2r A 141 SER	1.3					put isochorismatase YECD			2.85
1j9L A 93 GLY	1j9l A 94 VAL	1.9					"SURE" protein	stationary phase survival		-0.44
1jub B 191 SER	1jub B 192 VAL	1.4					dihydroorotate DH			1.64
1kb0 A 280 LEU	1kb0 A 281 TYR	1.44				b-prop	quino-heme alc DH	Y281 gd, alt 24 bad		1.04
1kms A 116 GLY	1kms A 117 GLY	1.09		GG			DHFR; NH bd NADP	end b, 117 N-cap		4.45
1m7a B 113 GLY	1m7a B 114 GLY	1.76		GG			Candida DHFR			1.95
1mdo A 329 HIS	1mdo A 330 PHE	1.7					ARNB aminotransferase			-0.36
1me8 A 290 GLY	1me8 A 291 ASN	1.9			?		inosine mono-P DH			0.82
1n0w A 222 ASP	1n0w A 223 SER	1.7					Rad51-BRCA2	DNA repair		1.13
1nf8 A 150 VAL	1nf8 A 151 TYR	1.6					isochorismatase	phenazine biosynthesis		-8.83
1nLf A 140 ASP	1nlf A 141 THR	1.95					DNA helicase RepA			-0.51
1nls A 207 ALA	1nls A 208 ASP	0.94				lectin	concanavalin A	this 1 gd, 2 awful		0.9
1nnw B 41 GLY	1nnw B 42 ASN	1.9					hyp prot Pfu-1218608			-2.59
1nth A 234 ASN	1nth A 235 GLU	1.55	2				monomethylene meth tr'ase			-1.35
1nth A 366 GLY	1nth A 367 VAL	1.55	2				"	act site, makes pyrrolysine !		1.62
1nuL A 36 SER	1nuL A 37 ARG	1.8					XG P-ribosyltransferase			-1.28
1nxm A 74 GLY	1nxm A 75 LEU	1.3			?	jellyroll	DTDP dehydrorhamnose epimerase			-0.53
1o1z A 192 TRP	1o1z A 193 THR	1.6		Wx	?	TIM	GDPD phosphodiesterase	str 8		-23.47
1o2d A 138 ALA	1o2d A 139 GLY	1.3					alcohol DH, Fe containing			-4.09
1o54 A 107 THR	1o54 A 108 GLY	1.65					SAM-dep O-methyltransferase			-14.99
1odo A 293 PRO	1odo A 294 LEU	1.85					put cytochrome P450			0.04
1ofs C 80 ALA	1ofs C 81 ASP	1.8				lectin	legume lectin			-0.8
1ofz A 273 ASN	1ofz A 274 ILE	1.5	2			b-prop	fungal lectin, in lps			2.76
1ofz A 284 GLY	1ofz A 285 VAL	1.5	2			lectin	"	fucose-specific		-5.07
1oi2 A 69 MET	1oi2 A 70 HIS	1.75					dihydroacetone kinase			4.79
1oi6 B 61 GLY	1oi6 B 62 ILE	1.4			?		DTDP deoxy-D-glucose epimerase			-2.57
1oi7 A 120 GLY	1oi7 A 121 GLY	1.23		GG			succinyl coA synthase	bulges end of par b,~ buried		0.77
1ong A 166 GLU	1ong A 167 THR	1.1					b-lactamase SHV-1			2.9
1oq1 B 135 SER	1oq1 B 136 ARG	1.7				lectin	hyp prot, galectin fold			-0.21
1q0q A 184 THR	1q0q A 185 GLY	1.9			?		deoxy-D-xylulose-P red'isomerase			6.36
1q7L A 113 ASP	1q7l A 114 MET	1.4					Zn-bdg aminoacylase			-1.08
1q8f A 12 PRO	1q8f A 13 GLY	1.7					pyrimidine nucleosidase YEIK			-13.6
1qb7 A 80 ASP	1qb7 A 81 ALA	1.5					Leishm adenine P-ribosyltr'ferase			0.69
1qgu A 251 TRP	1qgu A 252 SER	1.6		Wx			nitrogenase Mo-Fe (not TIM)			-8.49
1qyb A 78 GLY	1qyb A 79 ILE	1.75					rubrerythrin, Desulfovibrio vulg	at dimer contact		-0.45
1r3q A 303 GLY	1r3q A 304 ASN	1.7					uroporphyrinogen decarboxylase			-3.7
1rhc A 72 CYS	1rhc A 73 ILE	1.8				TIM	F420-dependent alcohol DH	luciferase family		-0.85
1rwj A 36 HIS	1rwj A 37 THR	1.7					3-heme cytochrome C7			-0.14
1ryi B 258 GLY	1ryi B 259 ALA	1.8					glycine oxidase			-4.93
1smo A 126 PHE	1smo A 127 ASP	1.47					triggering receptor	human innate imm		-6.22
1t0b D 157 GLU	1t0b D 158 PHE	1.7					THUA-like prot	trehalose metab		6.38
1t8h A 40 ALA	1t8h A 41 SER	1.8					hyp prot, B stear	YLMD homolog		-4.17
1t8t B 159 GLY	1t8t B 160 VAL	1.85					heparan sulfotransferase			-1.88
1thz A 431 SER	1thz A 432 ASN	1.8					lipase			-1.02
1u0f A 383 GLY	1u0f A 384 THR	1.6			?		phosphoglucose isomerase			4.52
1u94 A 144 ASP	1u94 A 145 SER	1.9					Rec-A, E coli	DNA repair		0.05
1ua4 A 28 GLY	1ua4 A 29 TYR	1.9			?		ADP-dep glucokinase			-0.09
1uek A 234 GLY	1uek A 235 SER	1.7					cytidine-di-P erythritol kinase			8.2
1usg A 75 GLY	1usg A 76 HIS	1.53					Leu-bdg prot	transport		-9.09

1uuf A 337 LYS	1uuf A 338 TYR	1.76					Zn alcohol-DH-like prot		0.13
1v5v B 279 GLU	1v5v B 280 VAL	1.5	2				T protein, Pyr hor	Gly-cleavage system	0.66
1v5v B 82 ALA	1v5v B 83 ILE	1.5	2				"		-0.72
1vch C 60 GLU	1vch C 61 THR	1.94					P-ribosyltransferase-related		0.96
1vd6 A 192 TRP	1vd6 A 193 THR	1.3		Wx	?	TIM	GDPD phosphodiesterase	str 8	-4.05
1vhe A 182 ASP	1vhe A 183 ASN	1.9					aminopeptidase/glucanase homolog		0.42
1vhv A 217 PRO	1vhv A 218 LEU	1.75					diphthine synthase		0.99
1vLr A 268 TYR	1vLr A 269 LEU	1.83					mRNA decapping enzyme		-0.21
1vme B 150 LEU	1vme B 151 HIS	1.8					TM0755 flavoprotein		17.11
1vpd A 231 PRO	1vpd A 232 GLY	1.65					tartronate semialdehyde reductase		-4.1
1vzi B 63 VAL	1vzi B 64 GLY	1.15					superoxide reductase		-5.63
1wko A 86 LYS	1wko A 87 GLU	1.8					terminal flower 1, arabidopsis		-3.25
1wog E 118 GLY	1wog E 119 GLY	1.8		GG			Dein rad agmatinase		0.61
1xbu A 97 ASP	1xbu A 98 ASN	1.2					strep aminopeptidase, Zn		1.84
1xpm D 308 GLY	1xpm D 309 SER	1.6					HMG-coA synthase		0.14
1xtt C 79 LEU	1xtt C 80 ARG	1.8					uracil P-ribosyltransferase		0.49
1y07 C 65 VAL	1y07 C 66 GLY	1.55				all-beta	superoxide reductase, Fe		-5.43
1y0b D 58 GLU	1y0b D 59 SER	1.8					xanthine P-ribosyltransferase		-0.85
1y7w A 219 PRO	1y7w A 220 GLY	1.86					halotolerant carbonic anhydrase		2.81
1yac B 113 VAL	1yac B 114 VAL	1.8					YCAC 8-mer hydrolase		-0.3
1ydy A 313 TYR	1ydy A 314 THR	1.7			?	TIM	glycero- P-diesterase		-4.48
1ye8 A 106 ASP	1ye8 A 107 GLU	1.4					put kinase-like prot		11.66
1ykd A 154 PHE	1ykd A 155 ASP	1.9	2				GAF doms adenyl cyclase	cyaobact; binds ribose	3.18
1ykd A 341 PHE	1ykd A 342 ASP	1.9	2				"		-6.42
1yo3 A 46 PRO	1yo3 A 47 THR	1.65					dynein light chain		6.32
1yoe A 11 PRO	1yoe A 12 GLY	1.78					pyrimidine nucleosidase		-10.94
1yqd A 346 ARG	1yqd A 347 TYR	1.65					synapyl alcohol DH, aspen		0.85
1ys1 X 292 GLN	1ys1 X 293 LEU	1.1					triacylglycerol lipase		-7.86
1z2n X 82 LEU	1z2n X 83 GLU	1.2			?		inositol 1,3,4-tris-P kinase		7.49
1z9t A 32 ASP	1z9t A 33 SER	1.54					put laccase		-1.9
1zn8 A 65 ASP	1zn8 A 66 SER	1.76					adenine P-ribosyltransferase		4.45
1zu3 A 9 PRO	1zu3 A 10 HIS	1.33					scorpion neurotoxin		-1.41
2a0m A 119 GLY	2a0m A 120 GLY	1.6		GG			trypanosome arginase		2.44
2ad6 A 269 LYS	2ad6 A 270 TRP	1.5					methanol DH		2.48
2aee B 74 ALA	2aee B 75 THR	1.95					orotate P-ribosyltransferase		-5.32
2asc A 10 ASN	2asc A A 11 TYR	1.1	altA,B				scorpion toxin	gd - just sc alts	-11.63
2b7r A 175 ALA	2b7r A 176 TRP	1.7					fumarate reductase flavoprotein		-9.22
2bcr A 508 GLY	2bcr A 509 SER	1.75					DNA polymerase lambda		1.05
2bw0 A 228 ASP	2bw0 A 229 LYS	1.7					formyl THF DH		14.28
2c0z A 60 GLY	2c0z A 61 ILE	1.6			?		keto-deoxy-sugar epimerase		-6.69
2c61 A 247 THR	2c61 A 248 ASP	1.5					non-catalytic ATPase subunit B		2.7
2c6q B 157 GLY	2c6q B 158 ASN	1.7					guanosine mono-P reductase		13.55
2d52 B 392 GLY	2d52 B 393 ILE	1.6					pentaketide chromone synthase		-0.91
2dg1 C 232 HIS	2dg1 C 233 GLU	1.72					drug-responsive prot	lactonase?	0.72
2dur A 130 GLY	2dur A 131. ASP	1.65			?	lectin	membrane VIP36, mannose-bdg	b-sandwich	-1.54
2dy0 A 64 GLU	2dy0 A 65 ALA	1.25					adenine P-ribosyltransferase		-8.15
2eja B 277 GLY	2eja B 278 ASN	1.9					uroporphyrinogen decarboxylase		0.32
2f5t X 186 TYR	2f5t X 187 LYS	1.45	1 gd		?		CHO transcr factor	2 passed, S-I 211 Dire Geom	0.47
2f7v A 103 ASP	2f7v A 104 ILE	1.75					acetylcitrulline deacetylase		-0.38
2fmp A 274 GLY	2fmp A 275 SER	1.65					DNA polymerase beta		0.4
2fn3 A 277 VAL	2fn3 A 278 PHE	1				dbl-wd	benzylformate de-CO-ase	buried; turn well past b	-21.22
2fnu A 315 HIS	2fnu A 316 TYR	1.5					PSEC aminotransferase		5.91
2fo3 A 76 GLN	2fo3 A 77 LYS	1.86					ubiquitin-conj E2		-4.67
2g0w A 76 GLU	2g0w A 77 TYR	1.7			?		put sugar isomerase		-1.4
2gfq B 156 GLY	2gfq B 157 SER	1.75					hypoth prot, P hor		7.54
2gh9 A 60 VAL	2gh9 A 61 THR	1.95			?	lectin	maltotriose-bdg prot		-2.87
2gi3 A 140 GLY	2gi3 A 141 SER	1.8					Gln-tRNA amidotr'ase		1.73
2h88 A 401 ALA	2h88 A 402 SER	1.74					succinate DH cytochrome B sub		-2.39
2hmc A 45 TYR	2hmc A 46 CYS	1.9					dihydrodipicolinate synthase		-4.08
2hrz A 190 CYS	2hrz A 191 ILE	1.85			?		nucleoside-di-P-sugar epimerase		0.66
2hxv A 282 GLY	2hxv A 283 GLY	1.8		GG			TM1828 new enzyme?	~DHFR, very like 3nrr	-1.04
2i71 B 83 GLU	2i71 B 84 SER	1.7					conserved hypoth prot		-1.35
2i7g B 79 ALA	2i7g B 80 VAL	1.73				TIM	mono-oxygenase		16.2
2ic7 B 65 PRO	2ic7 B 66 ASN	1.78			?		maltose transacetylase		-0.6
2imh B 103 ASP	2imh B 104 ILE	1.57					hypoth prot		-6.11
2imq X 224 TYR	2imq X 225 LYS	1.3					nitrophorin, Cimex		-5.97
2ixc A 60 GLY	2ixc A 61 LEU	1.79			?		CHO epimerase RmLc		-5.35
2ixk A 63 GLY	2ixk A 64 LEU	1.7			?		DTDP deoxy-D-glucose epimerase	lipopolysacch biosynth	0.02
2jae B 230 MET	2jae B 231 MET	1.25					L-amno-acid oxidase		4.39
2jdi A 269 ASP	2jdi A 270 ASP	1.9	2	DD			F1-ATPase	bovine heart mitochondria	1.54
2jdi D 256 ASP	2jdi D 257 ASN	1.9	2				"		14.21

2jis A 311 LEU	2jis A 312 GLN	1.6					Cys sulfinic acid decarboxylase		-10.97
2nrk A. 91 LYS	2nrk. A 92 GLY	1.65					conserved hypoth prot		5.7
2obL A 266 ASP	2obl A 267 SER	1.8					ATPase		11.99
2omz A 194 GLY	2omz A 195 ASN	1.6					internalin/E-cadherin		3.21
2opw A 104 GLY	2opw A 105 HIS	1.9					phytanoyl-coA dioxygenase		7.01
2oqx A 195 ILE	2oqx A 196 THR	1.9					tryptophanase		-2.77
2ozL A 133 GLY	2ozl A 134 GLY	1.9		GG			pyruvate DH E1	bulge end of parallel b str	1.31
2p0a B 192 VAL	2p0a B 193 ASN	1.9					synapsin III		-9.78
2phx B 85 ALA	2phx B 86 ASP	1.8				lectin	legume lectin		0.15
2piy B 197 SER	2piy B 198 TYR	1.43	3				carboxypeptidase B	b str end	-1.36
2piy B 205 PRO	2piy B 206 TYR	1.43	3				"		0.73
2piy B 272 ARG	2piy B 273 ASP	1.43	3				"		-5.03
2pok B 123 ASP	2pok B 124 ASP	1.9		DD			Mn peptidase		4.85
2prz D 74 ALA	2prz D 75 TYR	1.9					orotate P-ribosyltransferase		-3.44
2pwy A 100 ALA	2pwy A 101 GLY	1.7					tRNA methyltr'ase		-7.03
2q1u B 90 HIS	2q1u B 91 GLY	1.7			?		put nucleotide sugar epimerase		-5.31
2q7d A 94 LEU	2q7d A 95 ASP	1.6			?		inositol 1.3.4 tri-P kinase		-0.6
2q9u A 151 LEU	2q9u A 152 HIS	1.9					flavo-di-iron prot, giardia	b-lactamase-like	3.92
2qfs A 257 GLY	2qfs A 258 GLY	1.55		GG			EPSP synth, pesticide target		-1.24
2qia A 190 ASN	2qia A 191 HIS	1.74			?		N-acetylglucosamine epimerase		2.32
2qma B 792 ILE	2qma B 793 SER	1.81					Glu decarboxylase		-7.12
2qr6 A 198 GLY	2qr6 A 199 GLY	1.5			?		IMP DH/GMP red'ase	end par b (no related at 30%)	-2.59
2r84 B 205 SER	2r84 B 206 ASN	1.9					PURP ribonuceotide synthetase		5
2rau A 30 GLU	2rau A 31 ASP	1.85					put lipase		2.08
2rb7 B 99 ASP	2rb7 B 100 ASP	1.6		DD			peptidase		9.86
2rik A 123 PRO	2rik A 124 GLU	1.6					titin fragment		-2.4
2uzj B 36 GLN	2uzj B 37 SER	1.55					Cys protease	papain-like	1.21
2v4n A 90 GLY	2v4n A 91 ILE	1.7					"SURE" protein	stationary phase survival	-5.12
2v5z A 397 CYS	2v5z A 398 TYR	1.6					monoamine oxidase		0.35
2vLf B 182 VAL	2vLf B 183 THR	1.89					SOCS6 SH2, Tyr kinase		-14.96
2vLi B 148 GLN	2vLi B 149 THR	1.95					tunicamycin-resist, P-tr'ase		-0.71
2vvp B 36 GLY	2vvp B 37 ALA	1.65					ribose-5-P isomerase		6.89
2w3v A 103 GLY	2w3v A 104 GLY	1.89		GG			Mycobact DHFR		-1.64
2w9h A 93 GLY	2w9h A 94 GLY	1.48		GG			Staph DHFR	94 hlx N-cap	9.86
2wnp F 253 ASP	2wnp F 254 CYS	1.21			?	lectin	M-ficolin, sialic-acid bdg		-3.09
2wns A 75 PRO	2wns A 76 TYR	1.9					orotate P-ribosyltransferase		0.33
2wsd A 500 TYR	2wsd A 501 ASP	1.6					COTA laccase (Cu)		-0.21
2wta A 154 ILE	2wta A 155 ALA	1.7					nicotinamidase		-10.44
2wuu A 209 PRO	2wuu A 210 THR	1.42					myristoyltansferase		-8.9
2wwf C 208 LEU	2wwf C 209 TRP	1.89					thymidylate kinase		1.23
2x3c A 267 PRO	2x3c A 268 TYR	1.99					Zn metalloendopeptidase	~Cpase?	-1.83
2x3e B 312 GLY	2x3e B 313 LEU	1.81					Zn metalloendopeptidase	ACP synthase III	-3.28
2xio A 111 GLY	2xio A 112 GLU	1.19					put DNAse; GQ ed good	E112 in edo site w/ 3 His	3.63
2xjq A 160 ASP	2xjq A 161 ASP	1.35			?	lectin	flocculin, adhesin dom	binds CHOs	10.61
2xq0 A 186 GLU	2xq0 A 187 ALA	1.96					leukotriene aminopeptidase		3
2yvt A 40 GLY	2yvt A 41 ASN	1.6					hypoth prot		1.9
2yzk B 66 ALA	2yzk B 67 THR	1.8					orotate P-ribosyltransferase		24.7
2z0j E 25 ILE	2z0j E 26 ARG	1.5					conserved hypoth prot		1.31
2z2y A 313 PRO	2z2y A 314 ASP	1.89					Tk subtilisin		-7.95
2zbx A 142 PRO	2zbx A 143 THR	1.5					vit D hydroxylase cyt P450		-3.61
2zed A 159 ASP	2zed A 160 ALA	1.7					Gln-tRNA synthase		-4.64
3a2q A 149 GLY	3a2q A 150 SER	1.8					nylon oligomer degrading		0.93
3a2z A 56 LYS	3a2z A 57 TRP	1.5					glutathionylspermidine synthase		-10.61
3a4u A 120 ALA	3a4u A 121 ASP	1.84			?	lectin	ER-Golgi, mannose-bdg lectin		-6.03
3a57 A 64 LYS	3a57 A 65 TRP	1.5					thermostable direct hemolysin		-2.24
3a74 B 312 PRO	3a74 B 313 GLU	1.8					Lys-tRNA synthase		0.03
3a9s B 307 GLN	3a9s B 308 ARG	1.6			?		D-arabinose isomerase		0.08
3acd A 45 LEU	3acd A 46 ASN	1.89					H-G P-ribosyltransferase		0.51
3apa A 28 GLY	3apa A 29 GLU	1.65			?	lectin	Family 28 CBM, Cel5a		-8.31
3b4y B 73 SER	3b4y B 74 VAL	1.95					prob F420-dep glucose-6-P DH		-10.47
3b7s A 136 GLN	3b7s A 137 ALA	1.47					leukotriene hydrolase		-4
3bdv A 169 PHE	3bdv A 170 GLY	1.66					YDEN-like prot		10.63
3bec A 211 GLY	3bec A 212 ILE	1.6					penicillin-bdg, DD-peptidase		4.64
3bwx A 264 HIS	3bwx A 265 ALA	1.5				TIM	alpha/beta hydrolase		-5.47
3c6c A 199 LEU	3c6c A 200 TRP	1.72					keto-aminohexanoate cleavage		4.53
3ccz B 688 CYS	3ccz B 689 THR	1.7					hyd-methylglutaryl-coA reductase		-9.34
3ch0 A 241 TRP	3ch0 A 242 THR	1.5		Wx	?	TIM	phosphodiesterase	act site, 3 to hlx, str 8	-20.98
3cin A 206 GLY	3cin A 207 ASP	1.7			?		myo-inositol-1-P synthase reated		-4.18
3cjp B 120 GLY	3cjp B 121 GLU	1.85					put amidohydrolase	at Zn site	1.58
3cp7 B 192 THR	3cp7 B 193 PHE	1.39					extremophile Ser protease		10.73
3cq5 B 313 GLY	3cq5 B 314 ASP	1.8					histidinol-P transaminase	at surface	5.1

3d43 B 171 GLY	3d43 B 172 TYR	0.8					subtilisin B sphaericus	past str end, not at act site	13.18
3d4u A 197 SER	3d4u A 198 TYR	1.7	3				clot-lysis inhib, Met Cpase		-0.34
3d4u A 205 PRO	3d4u A 206 TYR	1.7	3				"		2.93
3d4u A 272 ARG	3d4u A 273 ASP	1.7	3				"		-1.71
3d59 A 72 PHE	3d59 A 73 ASP	1.5					platelet activating-F acetohydrolase		-10.74
3das A 338 ASP	3das A 339 ASP	1.6			?		aldose sugar DH		-9.33
3dau A 95 GLY	3dau A 96 GLY	1.5		GG			E coli DHFR	96 hlx N-cap	5.22
3dbk A 63 TYR	3dbk A 64 LYS	1.4					elastase with phosphoramidon	Pseudomonas	-0.15
3drw B 11 ALA	3drw B 12 TYR	1.9			?		phosphofructokinase		11.37
3dzw A 98 GLY	3dzw A A 99 THR	1.7	altA,B		?	lectin	Narcissus lectin	gd - just sc alts	0.26
3e2v B 184 GLY	3e2v B A 185 GLU	1.5	altA,B			TIM	amido hydrolase	gd - just sc alts	-1.38
3e8t A 40 ASP	3e8t A 41 VAL	1.3					insect takeout-like protein 1	transport	9.69
3ebh A 319 GLU	3ebh A 320 ALA	1.65					M1 Ala-aminopeptidase		-2.06
3ed4 B 240 GLU	3ed4 B 241 VAL	1.7					put arylsulftase		4.47
3eg4 A 114 PRO	3eg4 A 115 ASN	1.87				b-hlx	tetrahydropicolinate succinylase		6.78
3eif A 478 GLY	3eif A 479 LEU	1.9					C5a peptidase		0.61
3ejk A 66 ALA	3ejk A 67 TRP	1.95			?		DTDP sugar isomerase		-1.48
3ff1 B 301 TYR	3ff1 B 302 THR	1.65			?		glucose-6-P isomerase		-0.36
3fss A 243 GLY	3fss A 244 PHE	1.43					PH doms of histone chaperone		2.01
3fw3 A 201 PRO	3fw3 A 202 THR	1.72					carbonic anhydrase IV		8.67
3g0t B 238 PRO	3g0t B 239 LEU	1.75					put Asp aminotransferase		-1.76
3g7d A 410 HIS	3g7d A 411 LEU	1.8					"phpD": C-C cleavage	in phosphinothricin biosynth	-1.83
3gg7 A 83 GLY	3gg7 A 84 GLU	1.5					hypoth metalloprotein	at Mn site	1.54
3gmi A 84 PRO	3gmi A 85 GLY	1.91					hypoth protein		-4.82
3gwa A 334 GLY	3gwa A 335 TYR	1.6					oxoacyl ACP synthase		-5.65
3gyc B 332 CYS	3gyc B 333 TRP	1.85			?		put glycoside hydrolase		-9.53
3h12 B 144 SER	3h12 B 145 SER	1.5				TIM	put mandelate racemase		-0.78
3h78 A 318 GLY	3h78 A 319 ALA	1.7					PQSD quinolone signal biosynth		-6.77
3he8 B 33 GLY	3he8 B 34 THR	1.9					ribose-5-P isomerase		3.97
3hhs A 353 GLU	3hhs A 354 ALA	1.97	2				prophenyloxidase, insect	near Cu sites, hlx C-cap	-27.59
3hhs B 351 GLU	3hhs B 352 SER	1.97	2				"	(+1 wrong)	-18.33
3hoL A 351 CYS	3hol A 352 CYS	1.98		vic SS			transferrin-binding	vicinal SS (+2 wrong)	3.03
3hu5 A 134 THR	3hu5 A 135 GLN	1.5					isochorismatase		-1.97
3hyn A 28 HIS	3hyn A 29 ASP	1.2					put signal transduction prot		-0.48
3i1u A 197 SER	3i1u A 198 TYR	1.39	3				carboxypeptidase		-3.27
3i1u A 205 PRO	3i1u A 206 TYR	1.39	3				"		1.81
3i1u A 272 ARG	3i1u A 273 ASP	1.39	3				"		-6.64
3ife A 142 ASP	3ife A 143 ASP	1.55		DD			peptidase T		2.24
3iib A 292 ASP	3iib A 293 ASP	1.7		DD			peptidase		1.96
3iii A 444 PHE	3iii A 445 ASN	1.95			?		COCE/NOND family hydrolase		-7.09
3iL9 B 307 GLY	3iL9 B 308 PHE	1.85					E. coli FabH		-1.69
3imh A 70 GLY	3imh A 71 ARG	1.76			?		galactose-1 epimerase		9.43
3ip4 A 153 GLY	3ip4 A 154 SER	1.9					Glu/Asp-tRNA amidotransferase		1.08
3ipw A 112 GLY	3ipw A 113 GLU	1.95			?		TATD family hydrolase		-3.45
3irv A 174 THR	3irv A 175 VAL	1.6					Cys hydrolase PSPPH		-3.22
3isx A 168 ASP	3isx A 169 ASP	1.4		DD	?		endoglucanase		2.06
3iu1 A 288 PRO	3iu1 A 289 LYS	1.42					myristoyltransferase		-11.05
3k40 B 308 PHE	3k40 B 309 ASP	1.75					dihydroxyPhe decarboxylase		-7.94
3k7f B 216 GLY	3k7f B 217 SER	1.95					fatty acid amide hydrolase		10.76
3kgy B 150 GLY	3kgy B 151 GLY	1.5		GG			put DHFR		-3.88
3L12 A 273 TRP	3l12 A 274 THR	1.6		Wx	?	TIM	put phosphodiesterase	str 8	-13.31
3L5e B 283 TYR	3L5e B 284 ASP	1.53					beta-site amyloid cleaving enzyme		1.87
3L84 A 379 PHE	3L84 A 380 VAL	1.36					transketolase		6.36
3Led A 360 GLY	3Led A 361 TYR	1.45					oxoacyl-ACP synthase		-6.74
3Lez A 179 GLU	3Lez A 180 THR	1.25					halotolerant b-lactamase		-0.04
3LL9 B 172 PRO	3Ll9 B 173 GLY	1.85					put His kinase		5.09
3Ls9 A 330 ASN	3Ls9 A 331 ASP	1.4					atrazine chlorohydrolase TRZN		4.69
3mb5 A 100 ALA	3mb5 A 101 GLY	1.6					tRNA M1a58 methyltransferase		-10.57
3mcw B 120 VAL	3mcw B 121 SER	1.06				dbl-wd	isochorismatase hydrolase	end of b-str, Rossman	-9.01
3mhr A 105 SER	3mhr A 106 HIS	1.15					14-3-3 protein sigma		10.24
3mjd A 65 ALA	3mjd A 66 TYR	1.9					orotate P-ribosyl transferase		1.27
3mm6 A 129 PHE	3mm6 A 130 HIS	1.9	2				sulfite reductase		-3.5
3mm6 A 317 PHE	3mm6 A 318 VAL	1.9	2				"		-0.97
3n0x A 93 GLY	3n0x A 94 THR	1.5					branched amino-acid transporter		-8.91
3n5b B 37 GLN	3n5b B 38 ARG	1.9					signal transduction protein II		-3.52
3no3 A 222 TRP	3no3 A 223 THR	1.89		Wx	?	TIM	phosphodiesterase	str 8	-21.69
3nok A 61 HIS	3nok A 62 GLN	1.65				b-prop	Myxococcus Gln cyclase	~cPtt hp end of b-str	11.43
3nrr B 124 GLY	3nrr B 125 GLY	1.8		GG			tick-borne DHFR/thym synth	very like 2hxv	11.2
3nu8 B 308 HIS	3nu8 B 309 TYR	1.5			?		nucleotide sugar aminotransf'ase	O-antigen assembly	5.22
3o38 D 257 VAL	3o38 D 258 SER	1.95					short chain DH		5.18
3o3m D 35 PRO	3o3m D 36 VAL	1.82					hydroxyisocaproyll coA dehyd'ase		-3.08

3o7m D 41 LEU	3o7m D 42 LYS	1.98					HG P-ribosyltransferase		0.61
3o91 C 131 VAL	3o91 C 132 LEU	1.63					nicotinamidase		-9.05
3oa3 B 97 PHE	3oa3 B 98 HIS	1.6					put deoxyribose-P aldolase		-0.29
3oep A 127 ALA	3oep A 128 GLU	1.75					hypoth prot TTHA988		3.36
3op7 A 161 ASN	3op7 A 162 ALA	1.7					PLP-dep aminotransferase		-4.71
3p2u B 163 SER	3p2u B 164 ASP	1.48					PhnP protein ?		1.48
3p5p A 348 TYR	3p5p A 349 SER	1.82					taxadiene (diterpene) synthase		-8.24
3p6L A 105 GLY	3p6L A 106 VAL	1.85			?	TIM	sugar P isomerase/epimerase	str 3	6.49
3pb6 X 186 ASP	3pb6 X 187 SER	1.05				dbl-wd	Golgi-resident Gln cyclase	bulge in helix, D is Zn lig	3.15
3pfe A 129 ASP	3pfe A 130 ASP	1.5		DD			Zn peptidase		11.57
3pL2 B 165 ARG	3pL2 B 166 HIS	1.89					keto-deoxygluconokinase		-7.43
3pms A 204 CYS	3pms A 205 ALA	1.57					peptide: N-glycanase F		-4.9
3q62 B 71 HIS	3q62 B 72 PHE	1.4					hydroxydecanoyl-ACP dehyd'ase		-6.64
3q6j A 501 PHE	3q6j A 502 LEU	1.92					ketopropyl coenz M carboxylase		-2.66
3q7i A 372 GLY	3q7i A 373 THR	1.54					put ribose-5-P isomerase		12.22
3q7m A 114 GLY	3q7m A 115 VAL	1.65				b-prop	membrane lipoprotein, BAMB	[+ 4 wrong]	-0.65
3qd5 B 43 GLY	3qd5 B 44 VAL	1.9					put ribose-5-P isomerase		-3.5
3qki B 406 GLY	3qki B A 407 THR	1.92	altA,B		?		glucose-6-P isomerase	sc alts, only in ch B	4.77
