# Supplementary material for: Cis‐nonProline peptides: Genuine occurrences and their functional roles
Source: Protein Sci. 2025 May 24;34(6):e70157. doi: 10.1002/pro.70157 (PMC12102755; doi:10.1002/pro.70157)
Supplement: Supplementary file 3 — Table S3: A data table of the few residues which should have been cis‐nonPro but were not. [file PRO-34-e70157-s001.pdf]

Table-S3: Overlooked cis-nonPro in Top2018

PDB id	resolu	chain	1st res	2nd res	name
1h4p	1.75	A	Trp 409	Cys 410	beta glucanase
1p1j	1.7	A	Gly 318	Asp 319	inositol-1-P synthase
1r9d	1.8	A	Asp 527	Asn 528	glycerol dehydratase
1u6e	1.85	A	Gly 307	Leu 308	ACP synthase
1v08	1.9	A	Trp 457	Ser 458	maize beta-glucosidase
1xfk	1.8	A	Gly 124	Gly 125	formimino-glutamase
1xx1	1.75	C	Trp 230	Ser 231	sphingomyelinase D
2p0u	1.9	A	Gly 394	Leu 308	stilbenecarboxylase synthase
2yyv	1.65	A	Leu 23	Arg 24	uncharacterized prot, T maritima
3bh4	1.4	A	Trp 185	Glu 186	alpha-amylase
3bof	1.7	B	Asn 411	Ser 412	Met synthase
3dan	1.8	A	Pro 60	Phe 61	allene oxide synthase
3h49	1.8	A	Ala 140	Ser 141	putative ribokinase

Done for cis-nonPro as for cis-Pro, as in: Touw WG, Joosten RP, Vriend G (2015) Detection of trans-cis and peptide-plane flips in protein structures, Acta Crystallogr D71:1604-1614

For 1xx1, the fittings are wrong, but it is not clear that they are cis.

For 3bof, chain A is cis-nonPro but twisted. For all others that have two identical chains, both should be cis.
